# Supplementary material for: Neuroprotective and Disease-Modifying Effects of the Triazinetrione ACD856, a Positive Allosteric Modulator of Trk-Receptors for the Treatment of Cognitive Dysfunction in Alzheimer’s Disease
Source: Int J Mol Sci. 2023 Jul 6;24(13):11159. doi: 10.3390/ijms241311159 (PMC10342804; doi:10.3390/ijms241311159)
Supplement: Supplementary file 1 [file ijms-24-11159-s001.zip › ijms-2451828-supplementary.pdf]

Supplemental figure S1.

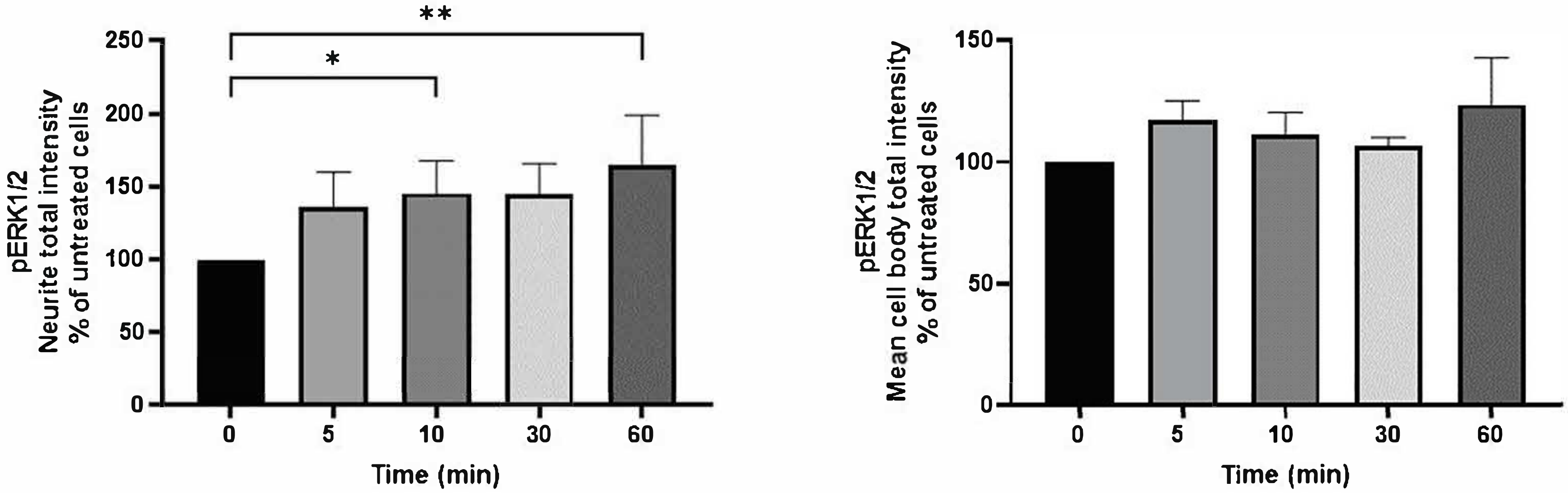

(a) (b)

Figure S1. The levels of phosphorylated ERK1/2 were determined by immunocytochemistry and neurite total intensity of pERK1/2-positive neurites (a) or the total intensity of pERK1/2 in cell bodies were determined (b). *$p < 0.05$, **$p < 0.01$ compared to control group at one-way ANOVA with Dunnett's multiple comparisons test. Data shown are the mean value of all replicates +/- SEM from three different experiments.
